# Conversion from epithelial to partial-EMT phenotype by *Fusobacterium nucleatum* infection promotes invasion of oral cancer cells

**DOI:** 10.1038/s41598-021-94384-1

**Published:** 2021-07-22

**Authors:** Wenhua Shao, Natsumi Fujiwara, Yasuhiro Mouri, Satoru Kisoda, Kayo Yoshida, Kaya Yoshida, Hiromichi Yumoto, Kazumi Ozaki, Naozumi Ishimaru, Yasusei Kudo

**Affiliations:** 1 Department of Oral Bioscience, Tokushima University Graduate School of Biomedical Sciences, Tokushima, 770-8504 Japan; 2 Oral Health Care Promotion, Tokushima University Graduate School of Biomedical Sciences, Tokushima, 770-8504 Japan; 3 Oral Health Care Education, Tokushima University Graduate School of Biomedical Sciences, Tokushima, 770-8504 Japan; 4 Periodontology and Endodontology, Tokushima University Graduate School of Biomedical Sciences, Tokushima, 770-8504 Japan; 5 Oral Molecular Pathology, Tokushima University Graduate School of Biomedical Sciences, Tokushima, 770-8504 Japan

**Keywords:** Bacteria, Pathogens, Oral cancer, Head and neck cancer, Oral cancer, Cancer, Microbiology, Pathogenesis

## Abstract

The ability of cancer cells to undergo partial-epithelial mesenchymal transition (p-EMT), rather than complete EMT, poses a higher metastatic risk. Although *Fusobacterium nucleatum* mainly inhabits in oral cavity, attention has been focused on the *F. nucleatum* involvement in colorectal cancer development. Here we examined the p-EMT regulation by *F. nucleatum* in oral squamous cell carcinoma (OSCC) cells. We cultured OSCC cells with epithelial, p-EMT or EMT phenotype with live or heat-inactivated *F. nucleatum*. Expression of the genes involved in epithelial differentiation, p-EMT and EMT were examined in OSCC cells after co-culture with *F. nucleatum* by qPCR. Cell growth and invasion of OSCC cells were also examined. Both live and heat-inactivated *F. nucleatum* upregulated the expression of p-EMT-related genes in OSCC cells with epithelial phenotype, but not with p-EMT or EMT phenotype. Moreover, *F. nucleatum* promoted invasion of OSCC cells with epithelial phenotype. Co-culture with other strains of bacteria other than *Porphyromonas gingivalis* did not alter p-EMT-related genes in OSCC cells with epithelial phenotype. *F. nucleatum* infection may convert epithelial to p-EMT phenotype via altering gene expression in OSCC. Oral hygiene managements against *F. nucleatum* infection may contribute to reduce the risk for an increase in metastatic ability of OSCC.

## Introduction

Head and neck cancer is the seventh most common cancer overall (the fifth most common in men and the 12th most common in women), accounting for an estimated 888,000 new cases in 2018^1^. Oral cancer, predominantly oral squamous cell carcinoma (OSCC), is a significant health problem and is considered the leading cause of death from oral diseases in many countries. According to GLOBOCAN (The global burden of cancer), it is estimated 354,864 new cases and 177,384 deaths of oral cancer in 2018^1^. Traditional risk factors of OSCC include alcohol consumption, tobacco and tobacco-derivate chewing and oral virus infections (HPV). Other factors include infections by poor oral hygiene, exposure to ionizing radiation, and environmental pollutants^2,3^. Thus, various causes of cancer are known to be closely involved in lifestyle choices such as smoking, drinking and diet. Inflammation caused by infections has been suggested to be one of the most important cause of cancers^4^. As more than 700 bacterial species exist in an oral cavity, bacterial infection may cause oral cancer. However, it remains unclear the role of bacteria in oral carcinogenesis even though a lot of bacteria inhabit in the oral cavity.

*Fusobacterium nucleatum* is an obligate Gram-negative anaerobic bacilli, which mainly inhabits in oral cavity^5^. It is associated with periodontal disease and pregnancy complications such as neonatal sepsis, still-birth and pre-term birth^6,7^. Moreover, it has been reported the association between *F. nucleatum* and colorectal^8,9^, esophageal^10^ and breast cancer^11^. Although there are many reports on the detection of *Fusobacterium* in OSCC tissues^12–14^, the detailed mechanism of *Fusobacterium* in OSCC cells remains unclear. The fact that *F. nucleatum* is abundant in the oral cavity of OSCC patients seems to be important in tumorigenesis and progression of oral cancer.

Epithelial mesenchymal transition (EMT) is a process by which epithelial cells lose their cell polarity and cell–cell adhesion, and gain mesenchymal properties. It can be seen during development, wound healing, inflammation, and cancer development. EMT contributes to cancer progression by imparting the mesenchymal phenotypes associated with the cells of highly aggressive tumors^15^. EMT is not binary process and cells move through between full epithelial and mesenchymal states, which is called the intermediate state of EMT, partial EMT (p-EMT)^16^. Cancer cells with p-EMT state act like cancer cells with mesenchymal state, but they do not completely lose their epithelial features^17^. We previously showed that p-EMT genes such as *SERPINE1*, *ITGA5*, *TGFBI*, *P4HA2*, *CDH13*, and *LAMC2* are potent poor prognostic markers in head and neck squamous cell carcinoma (HNSCC) patients by bioinformatic analysis^18^. Thus, understanding the state of p-EMT is crucial in finding a target cancer potential including cancer development and malignancy.

It recently has been reported that *F. nucleatum* is associated with EMT in cancer development. *F. nucleatum* decrease the levels of epithelial marker including E-cadherin and increase the levels of EMT-associated transcription factors including Snail and Slug in colorectal cancer progression^19^. Moreover, ZEB1 level is upregulated by *F. nucleatum* exposure in oral cancer cells^20^. However, the contribution of *F. nucleatum* to the regulation of p-EMT in OSCC remains unclear.

## Methods

### Cell culture

In this study, we used OSCC cell lines, HCS2, HOC313, HOC621, HOC719PE and HOC719NE. HSC2 cells were provided by the Japanese Collection of Research Bioresources Cell Bank. HOC313, HOC621, HOC719PE and HOC719NE cells were provided from Prof. Kamata (Hiroshima University). We previously found that HOC719NE and HOC313 have EMT features including loss of E-cadherin expression^21,22^. They were maintained in Dulbecco’s Modified Eagle Medium (Nacalai tesque, Inc., Kyoto, Japan) supplemented with 10% heat-inactivated FBS (Invitrogen) under conditions of 5% CO_2_ in air at 37˚C.

### Bacterial culture

*Fusobacterium nucleatum* (JCM8532, ATCC10953, JCM11024, and ATCC23726) and *Porphyromonas gingivalis* (ATCC33277) were cultured by brain heart infusion (BHI, Becton Dickinson, Sparks, MD, USA) supplemented with 5 µg/mL hemin and 0.5 µg/mL menadione anaerobically at 37 °C. *Streptococcus mutans* (UA159) was cultured by BHI anaerobically at 37 °C. *Pseudomonas aeruginosa* (PAO1) and *Escherichia coli* (ATCC23511) were grown using lennox L broth (LB, Thermo Fisher Scientific, MA, USA) with shaking at 37 °C. All bacteria were cultured to the absorbance at OD_590_ = 1.0 and used for following experiments.

For *F. nucleatum* inactivation, the bacterial cells were heat-inactivated at 100 °C for 15 min. Killing was confirmed by absence of growth on sheep blood agar plate (Becton Dickinson, Sparks, MD, USA). Other bacteria, *P. gingivalis, S. mutans, P. aeruginosa, and E. coli* were also heat-inactivated at 100 °C for 15 min.

To analyze the presence of bacteria, *F. nucleatum* were stained with CytoTell UltraGreen (AAT Bioquest, CA, USA) in accordance with the manufacturer’s instructions. Briefly, the bacterial cells were treated with CytoTell UltraGreen for 15 min at 37 °C, and then treated to HOC621 cells at the concentration of 100 MOI for 3 h. The cells were fixed with 4% formalin for 30 min and then permeabilized with 0.1% Triton X-100 in PBS for 2 min on ice, followed by Hoechst 33,342 for 30 min. The samples were mounted and observed by a Nikon A1 laser fluorescence confocal microscopy (A1 R HD25, Nikon, Tokyo). Images were acquired with the NIS-Elements software (Nikon, Tokyo, Japan).

### Colony-forming unit (CFU) determination

To quantify the viability of bacteria within host cells, colony-forming units were quantified as follows. OSCC cells were cultured on a 48-well plate, followed by treatment with *F. nucleatum* for 3 h, and then treated with antibiotics (200 μg/mL metronidazole and 300 μg/mL gentamicin) for 60 min to kill the *F. nucleatum* on the cell surface. The cells were lysed by adding 200 μL of H_2_O and the extract was spread on a sheep blood agar using a spiral plater (EDDY-Jet, IUL Instruments, Königswinter, Germany). Agar plates inoculated were incubated for 5 days in an anaerobic jar at 37 °C, and then, formed colonies were counted according to the manufacturer’s instructions.

### Cell proliferation assay

Cells were suspended 5,000 cells/well in 96-well plate. After 24, 48, and 72 h, proliferating cells were determined by using cell-counting kit 8 (CCK-8) solution (Dojindo Molecular Technique, Inc.). 10 µl of CCK-8 solution was added in each well and incubated for 1 h in CO_2_ incubator at 37 °C. The signal of proliferating cells was measured absorbance at 450 nm using microplate reader model 680 (BIORAD).

### qPCR

Total RNA was isolated from the cells by using the RNeasy Mini Kit (Qiagen) according to the manufacturer’s instructions. RNA concentration and purity were determined by standard spectrophotometric methods using a NanoDrop ND-2000 (NanoDrop Technologies). cDNA was synthesized from 1 µg total RNA using the PrimeScript II reverse transcriptase (Takara Bio Inc.). Expression of p-EMT, EMT and epithelial differentiation-relatedgenes were measured using TB Green Premix Ex Taq II (Takara Bio Inc.) with a LightCycler 96 system (Roche). The primers used in this study are shown in Supplementary Table S1. Relative mRNA expression of each transcript was normalized to the endogenous control GAPDH mRNA and were calculated using the comparative cycle threshold method.

Heatmap was generated by R package pheatmap. Expression values were centered and scaled in the row direction, each gene.

### In vitro invasion assay

For in vitro invasion assay, we used a 24-well cell culture insert with 8 μm pores (3097, Falcon, Becton Dickinson). The membrane of cell culture insert was coated with 20 μg of matrigel (Becton Dickinson) for reconstituting basement membrane substance. 0.5 ml of serum-free medium were added in the lower compartment. 1.5 × 10^5^ cells were resuspended in 100 μl of serum-free medium and placed in the upper compartment of the cell culture insert for 12–24 h. After incubation, the cells that penetrated the membrane into the lower side of the filter through the pores were fixed with formalin and then were stained by hematoxylin. The invasion ability of the cells was determined by counting the penetrating cells onto the lower side. We assayed 3 times. The penetrating cells in randomly selected 3 fields were counted for each assay under a microscope at × 100 magnification.

### Statistical analysis

For cell proliferation and qRT-PCR analyses, the *t*-test was used to compare results between two groups. A *P*-value of < 0.05 was considered significant.

## Results

**Figure 1:**
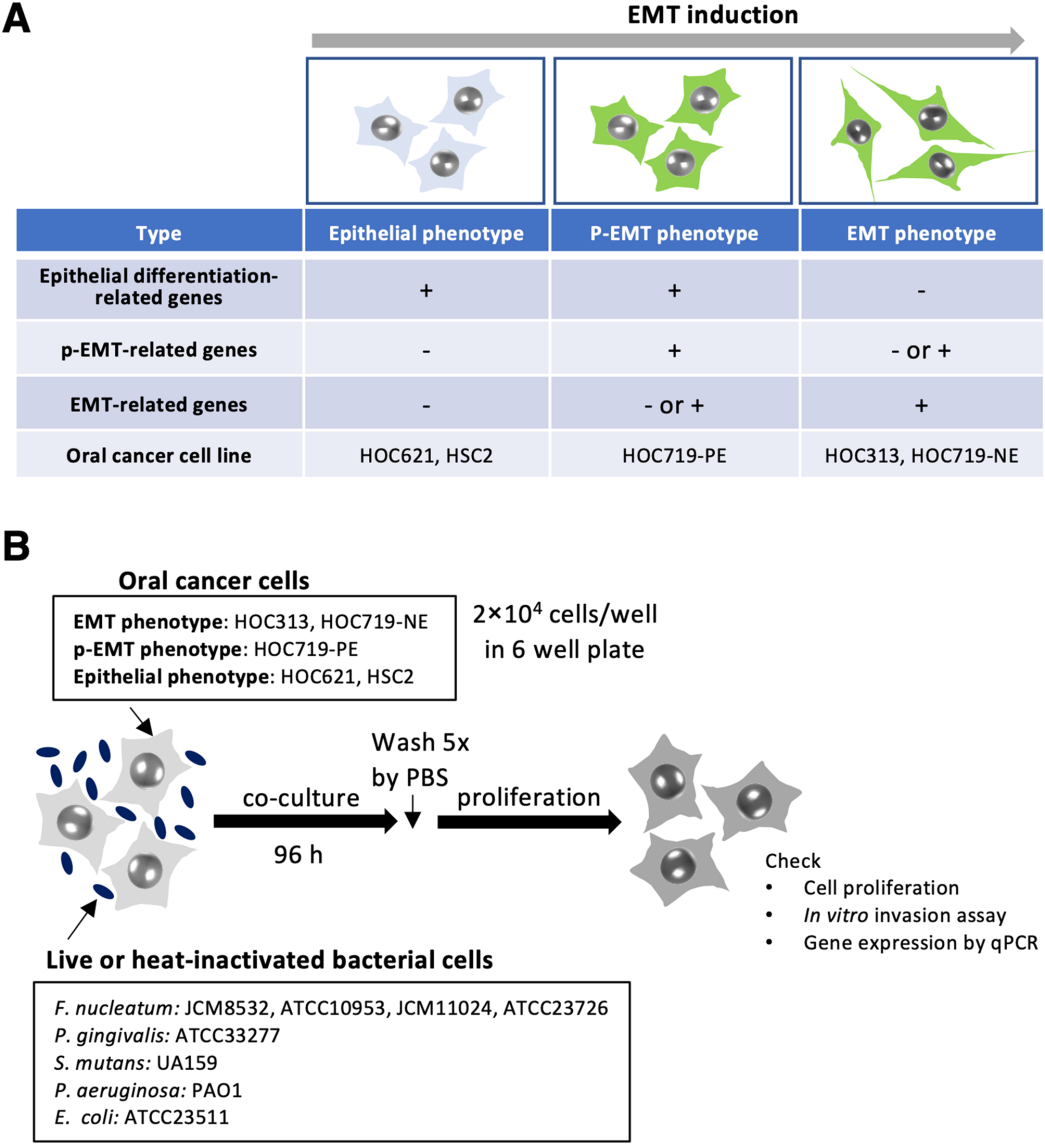
Strategies of this study. (**A**) The list of OSCC cell lines that showing their properties; epithelial phenotype, p-EMT phenotype, and EMT phenotype. The properties were defined by gene expression^18^. (**B**) Study flow of *Fusobacterium nucleatum* treatment to OSCC cells for analyses including the cell proliferation, in vitro invasion assay and gene expression. Four subspecies of *F. nucleatum* and 5 OSCC cell lines were used. After OSCC was co-cultured with *F. nucleatum* for 96 h and then washed by PBS five times. After washing, cells were used for the analyses.

**Figure 2:**
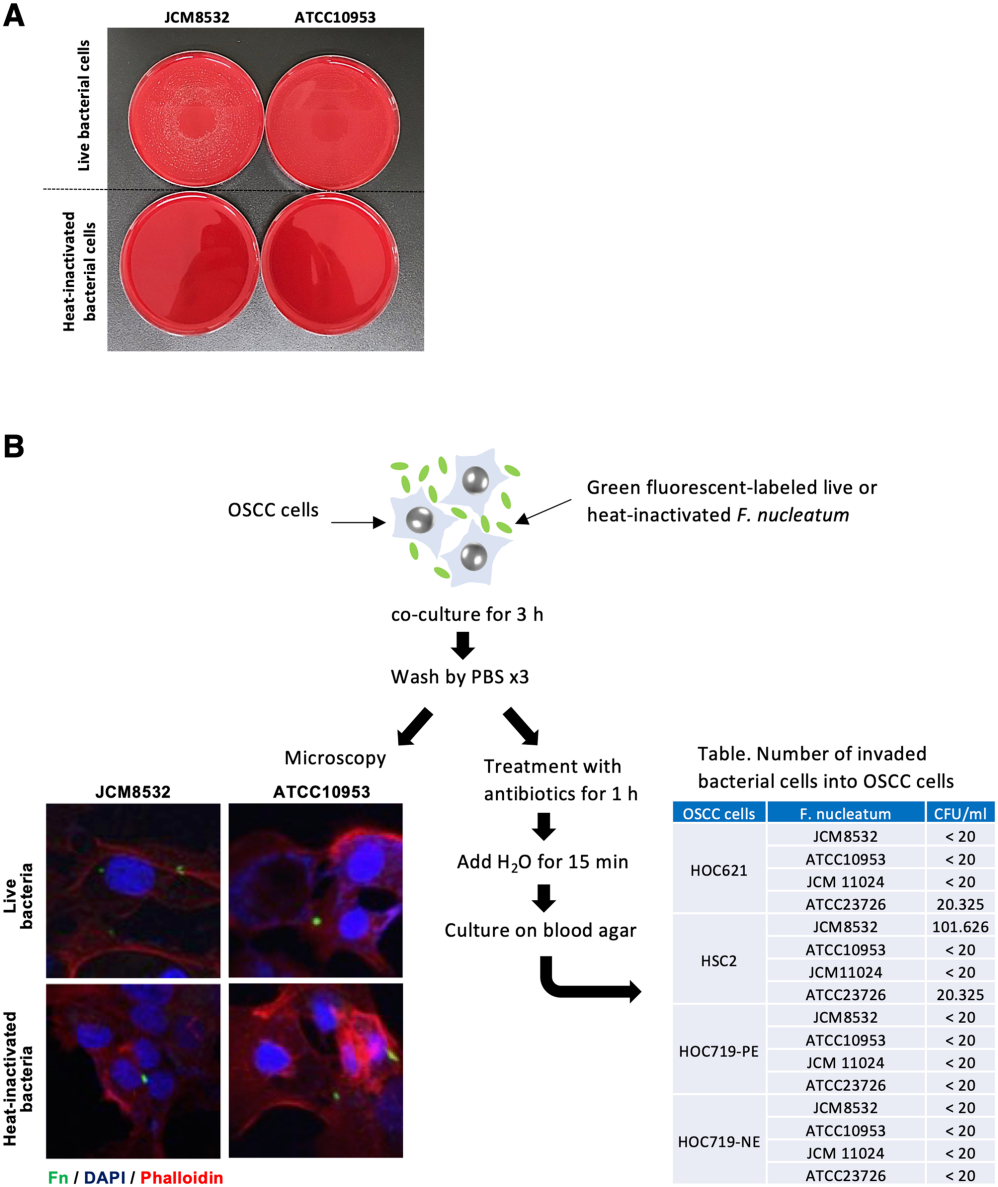
Preparation of heat-inactivated *F. nucleatum* and attachment of *F. nucleatum* on the surface of OSCC cells. (**A**) Pictures of *F. nucleatum* colonies. The heat-inactivated *F. nucleatum* bacterial cells were prepared by heating for 15 min at 100 °C. Bacterial cells show no colony forming on sheep blood agar after heat inactivation. (**B**) Attachment of *F. nucleatum* on the surface of OSCC cells. Green fluorescent-labeled live or heat-inactivated *F. nucleatum* (JCM8532 and ATCC10953) were co-culture with OSCC cells for 3 h. After washing by PBS, attached cells on the surface of OSCC cells were checked by immunofluorescence microscopy. Images show that heat-inactivated or live bacterial cells attach on the surface of OSCC cells. Moreover, invaded bacterial cells into OSCC cells were examined by culture on blood agar after treatment with antibiotics. Table shows the number of invaded *F. nucleatum* (CFU/ml) in OSCC cells (HOC621, HSC2, HOC719-PE, and HOC719-NE).

It recently has been shown that *F. nucleatum* plays a role in EMT induction of cancer cells. Moreover, the ability of cancer cells to undergo p-EMT poses a higher metastatic risk rather than complete EMT. We previously classified the OSCC cell lines as “epithelial”, “p-EMT”, and “EMT” phenotype by gene expression profiles (Fig. 1A). In this study, four subspecies of *F. nucleatum* (JCM8532, ATCC10953, JCM11024, and ATCC23726) and five OSCC cell lines (EMT phenotype, HOC313 and HOC719NE; p-EMT phenotype, HOC719-PE; epithelial phenotype, HOC621 and HSC2) were used (Fig. 1B). In our preliminary experiments, the effects of *F. nucleatum* at MOI = 10 and 100 on cell proliferation and gene expression was similar. Therefore, we used *F. nucleatum* at MOI = 10 in the following experiments. We cultured OSCC cells with live or heat-inactivated *F. nucleatum* for 4 days. As shown in Fig. 2A, we confirmed that bacterial cells after heating for 15 min at 100 °C did not grow on blood agar.

We first examined the attachment between OSCC cells and bacterial cells. HOC621 cells were co-cultured with green fluorescent-labeled live or heat-inactivated *F. nucleatum* (JCM8532 and ATCC10953) for 3 h, and then the cells were washed three times by PBS. After co-culture, live or heat-inactivated *F. nucleatum* was observed on the surface of HOC621 cells (Fig. 2B). We also examined the invasive potential of *F. nucleatum* (JCM8532, ATCC10953, JCM11024, and ATCC23726) into OSCC cells. After treatment with antibiotics, intracellular live bacterial cells were cultured on blood agar. Bacterial culture revealed that some bacterial cells invaded into OSCC cells (Fig. 2B).

**Figure 3:**
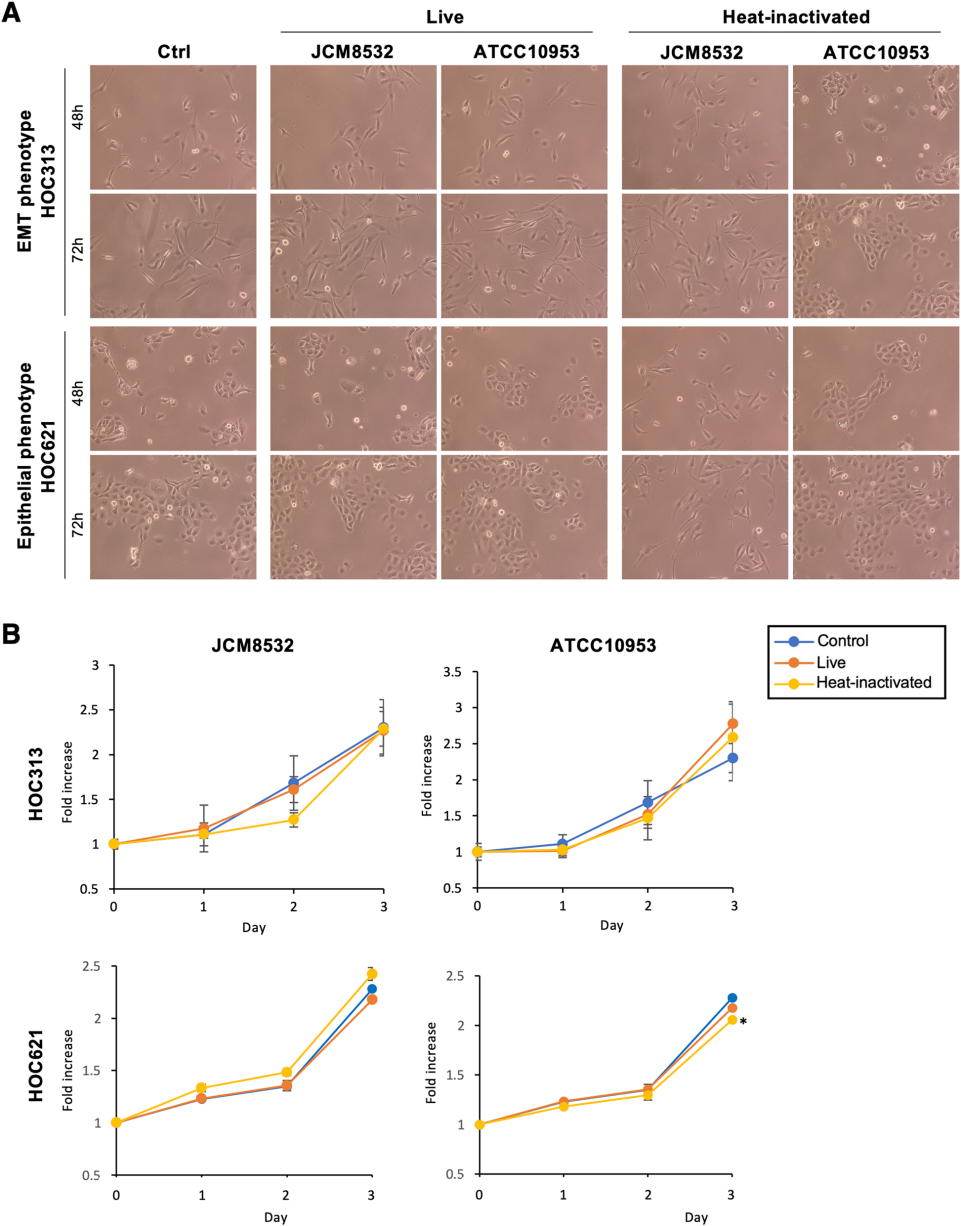
Cell morphology and proliferation in OSCC cells after co-culture with *F. nucleatum*. (**A**) Morphological images of OSCC cells after co-culture with *F. nucleatum* for 48 and 72 h. HOC313 and HOC621 were co-cultured with live or heat-inactivated *F. nucleatum* (JCM8532 and ATCC10953, MOI = 10). (**B**) Cell proliferation of HOC313 and HOC621 cells after co-culture with live or heat-inactivated *F. nucleatum* (JCM8532 and ATCC10953, MOI = 10). The graphs show the results as mean ± S.D. of three independent experiments. (*) represents *P*-value of < 0.05 compared to control (no treatment).

Then, we compared the effects of live or heat-inactivated *F. nucleatum* on cell morphology, proliferation, and gene expression in HOC313 cells (EMT phenotype) and HOC621 cells (epithelial phenotype). After 48 and 72 h, co-culture with live or heat-inactivated *F. nucleatum* did not change the morphology of OSCC cells (Fig. 3A). Co-culture with live *F. nucleatum*, except heat-inactivated *F. nucleatum* (ATCC10953), did not affect cell proliferation in both HOC313 and HOC621 cells (Fig. 3B). We also examined the cell proliferation of HOC621 cells after co-culture with other subspecies of *F. nucleatum* (JCM11024 and ATCC23726). Among them, co-culture with live or heat-inactivated *F. nucleatum*, except heat-inactivated *F. nucleatum* (ATCC23726), did not affect cell proliferation in HOC621 cells (Supplementary Fig. S1). Thus, co-culture with most *F. nucleatum* subspecies did not affect cell morphology and cell proliferation of OSCC cells.

**Figure 4:**
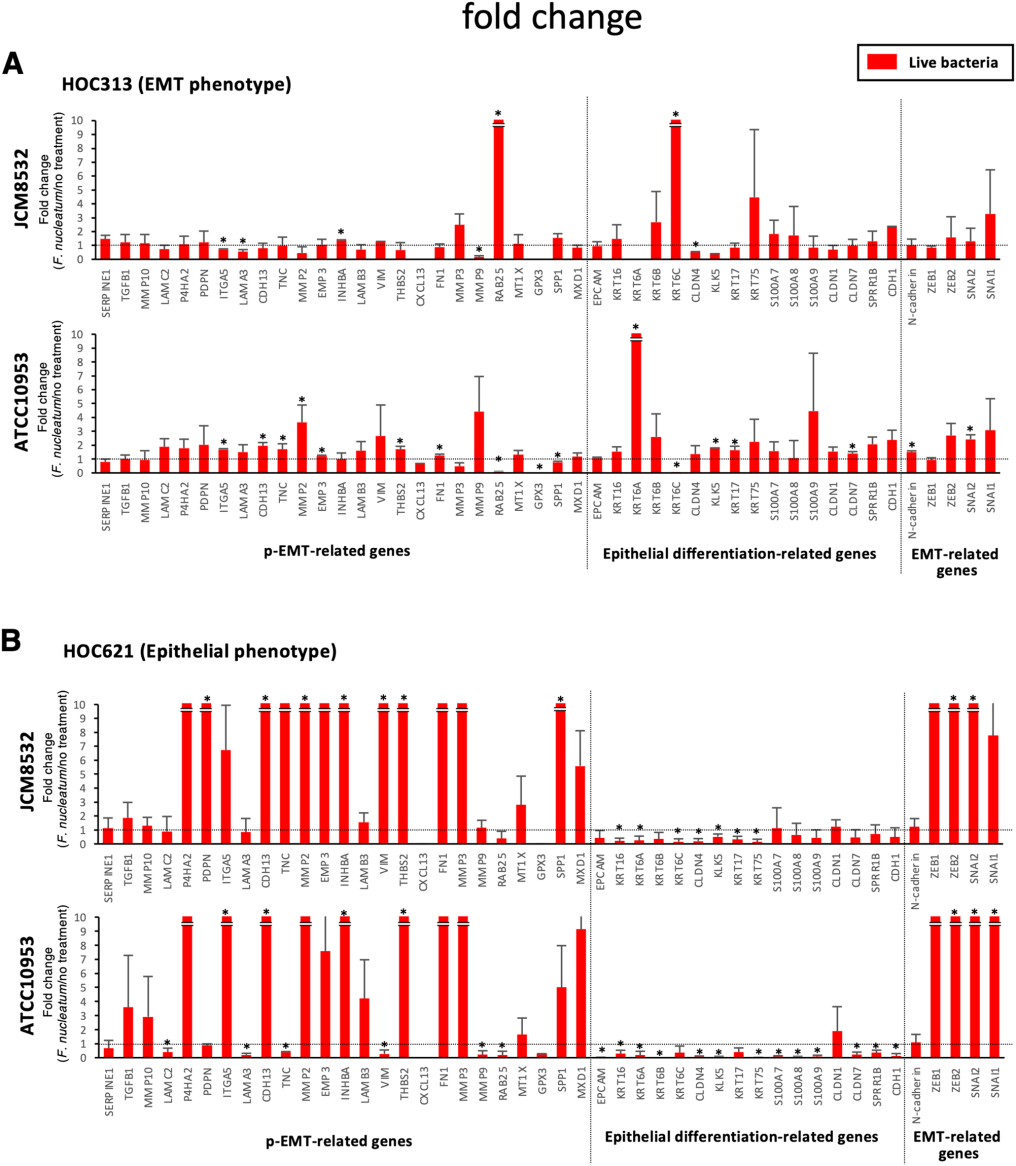
Co-culture with live *F. nucleatum* upregulates p-EMT-related genes in epithelial phenotype, HOC621 cells, but not in EMT phenotype, HOC313 cells. After co-culture with live *F. nucleatum* (JCM8532 and ATCC10953, MOI = 10), expression of epithelial differentiation-related, p-EMT-related, and EMT-related genes was determined by qPCR in EMT phenotype, HOC313 (**A**) and epithelial phenotype, HOC621 cells (**B**). The graphs show the results as mean ± S.D. of three independent experiments. (*) represents *P*-value of < 0.05 compared to control (no treatment).

Although *F. nucleatum* is associated with EMT in cancer development^19^, the involvement of p-EMT in *F. nucleatum* infection remains unclear. Therefore, we examined the involvement of *F. nucleatum* in the regulation of p-EMT of OSCC. Expression of epithelial differentiation-related genes, p-EMT-related genes, and EMT-related genes was examined by qPCR in HOC313 and HOC621 cells with live *F. nucleatum* (JCM11024 and ATCC23726). In HOC621 cells, but not HOC 313 cells, p-EMT-related and EMT-related genes were remarkably upregulated by co-culture with live *F. nucleatum* (Fig. 4A,B). Moreover, we confirmed similar tendency in other subspecies of *F. nucleatum* (JCM11024, and ATCC23726) in HOC621 cells (Supplementary Fig. S2). The upregulated levels of p-EMT-related genes depended on the bacterial subspecies, indicating that pathogenicity may vary depending on the bacterial subspecies. These findings suggest that live *F. nucleatum* may upregulate p-EMT genes in OSCC cells with epithelial phenotype.

**Figure 5:**
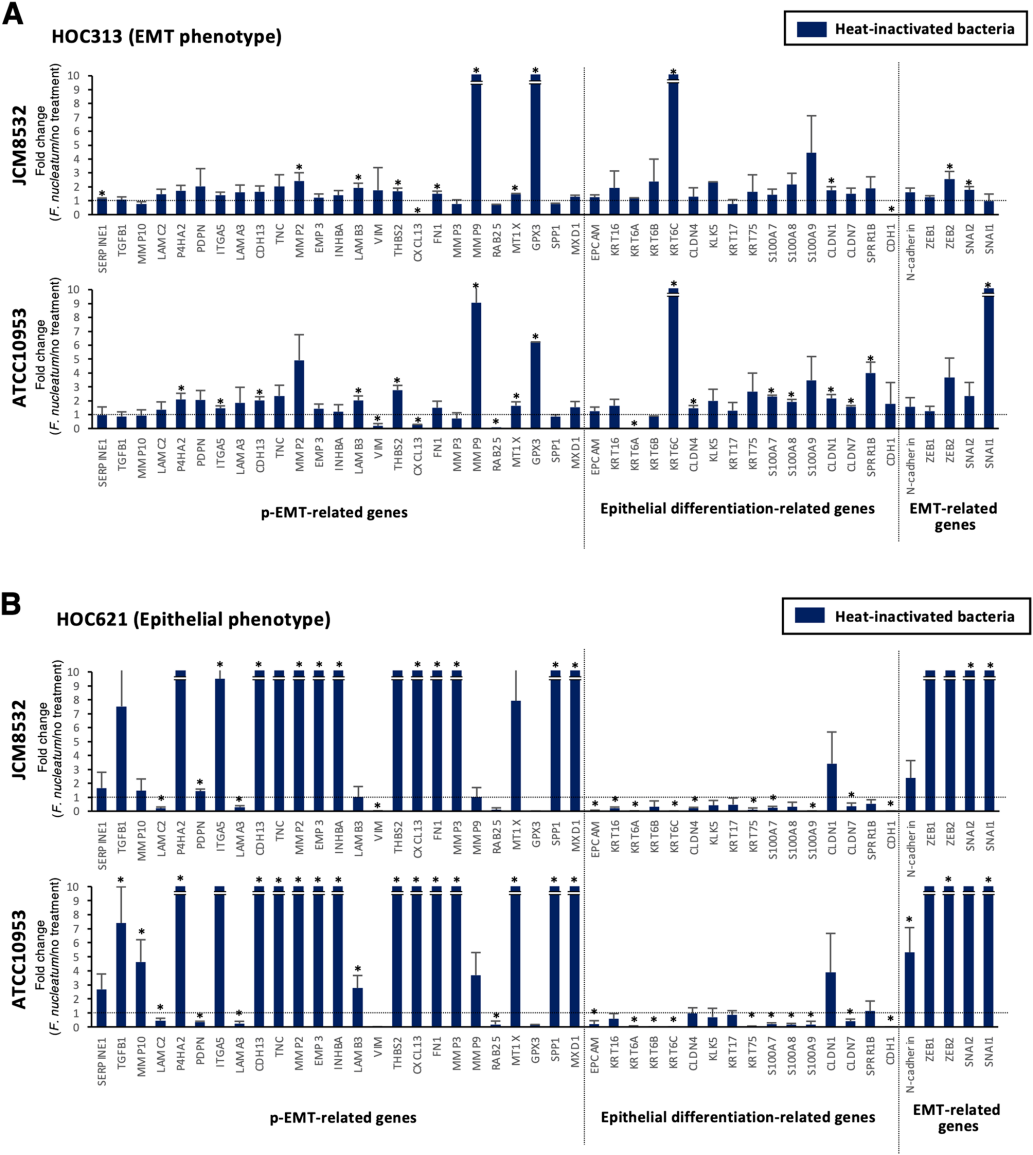
Co-culture with heat-inactivated *F. nucleatum* upregulates p-EMT-related genes in eEpithelial phenotype, HOC621 cells. After co-culture with heat-inactivated *F. nucleatum* (JCM8532 and ATCC10953, MOI = 10), expression of epithelial differentiation-related, p-EMT-related, and EMT-related genes was determined by qPCR in HOC313 (**A**) and HOC621 cells (**B**). The graphs show the results as mean ± S.D. of three independent experiments. (*) represents *P*-value of < 0.05 compared to control (no treatment).

**Figure 6:**
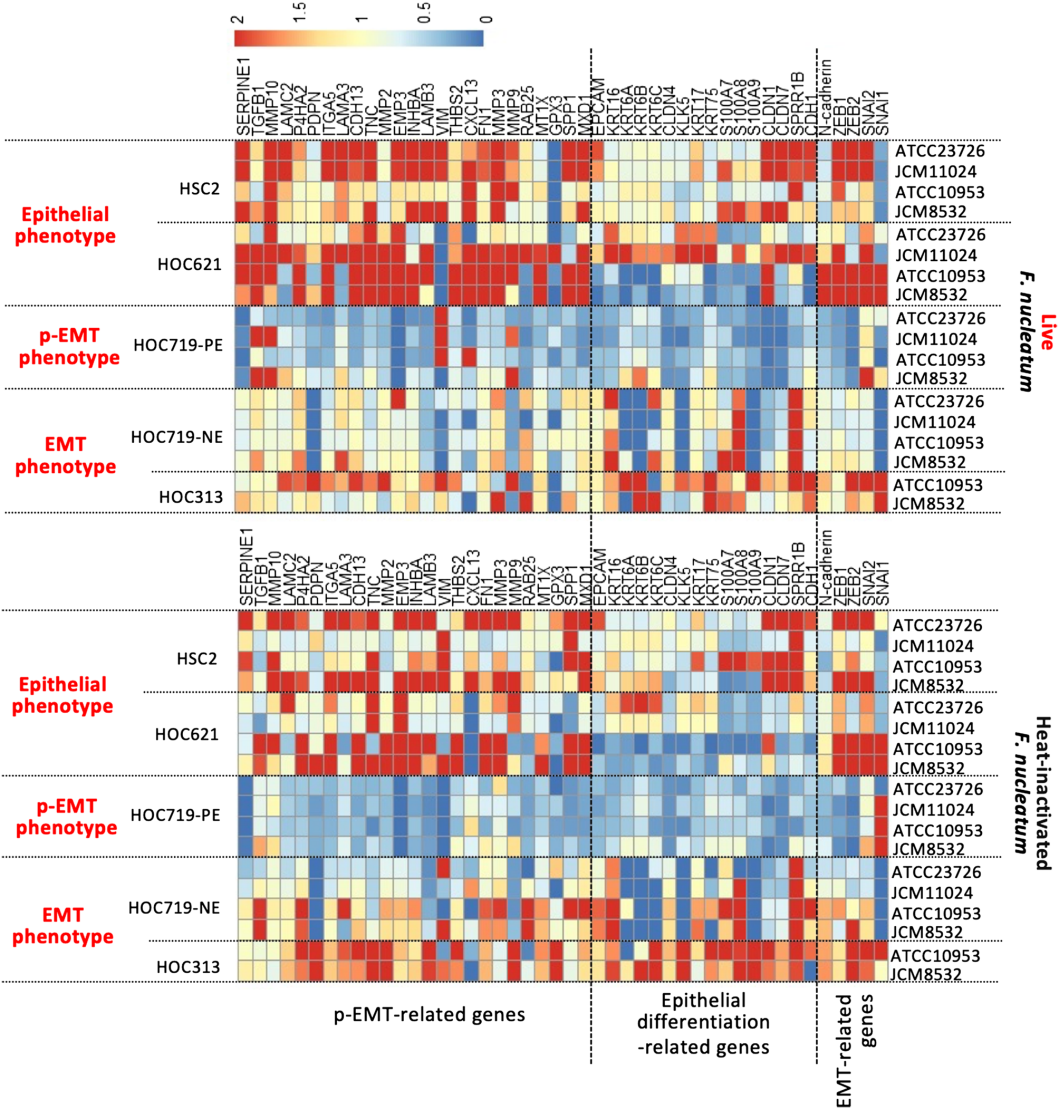
Heatmap of gene expression by co-culture with live or heat-inactivated *F. nucleatum* in OSCC cells. Heatmap shows live or heat-inactivated *F. nucleatum* (JCM8532, ATCC10953, JCM11024, and ATCC23726) in OSCC with epithelial (HOC621 and HSC2), p-EMT (HOC719-PE), and EMT phenotype (HOC313 and HOC719-NE). Gene expression data is shown in Fig. 4 (HOC313 and HOC621), Fig. 5 (HOC313 and HOC621), Supplementary Fig. S2 (HOC621), Supplementary Fig. S3 (HOC719-NE), Supplementary Fig. S4 (HSC2), and Supplementary Fig. S5 (HOC-719-PE).

Next, we examined whether the phenotype brought about by live *F. nucleatum* were also caused by heat-inactivated *F. nucleatum*. The heat-inactivated *F. nucleatum* (JCM8532 and ATCC10953) did not alter the expression of p-EMT-related genes in HOC313 cells (Fig. 5A). Interestingly, heat-inactivated *F. nucleatum* as well as live *F. nucleatum* upregulated p-EMT-related genes in HOC621 cells (Fig. 5B and Supplementary Fig. S2). Thus, both live and heat-inactivated *F. nucleatum* upregulated p-EMT-related genes in OSCC cells with epithelial phenotype, but not with EMT phenotype. To confirm these findings, we examined the gene expression in other OSCC cells with epithelial and EMT phenotype after co-culture with live or heat-inactivated *F. nucleatum* (Fig. 6). Alteration of p-EMT-related genes in epithelial phenotype, HSC2 cells (Supplementary Fig. S3) was remarkable after co-culture with live or heat-inactivated *F. nucleatum*, compared with EMT phenotype, HOC719-NE cells (Supplementary Fig. S4). We also examined the effect of live or heat-inactivated *F. nucleatum* in p-EMT phenotype, HOC719-PE cells. As expected, both live and heat-inactivated *F. nucleatum* did not alter the expression of p-EMT-related genes in HOC719-PE cells (Fig. 6 and Supplementary Fig. S5).

**Figure 7:**
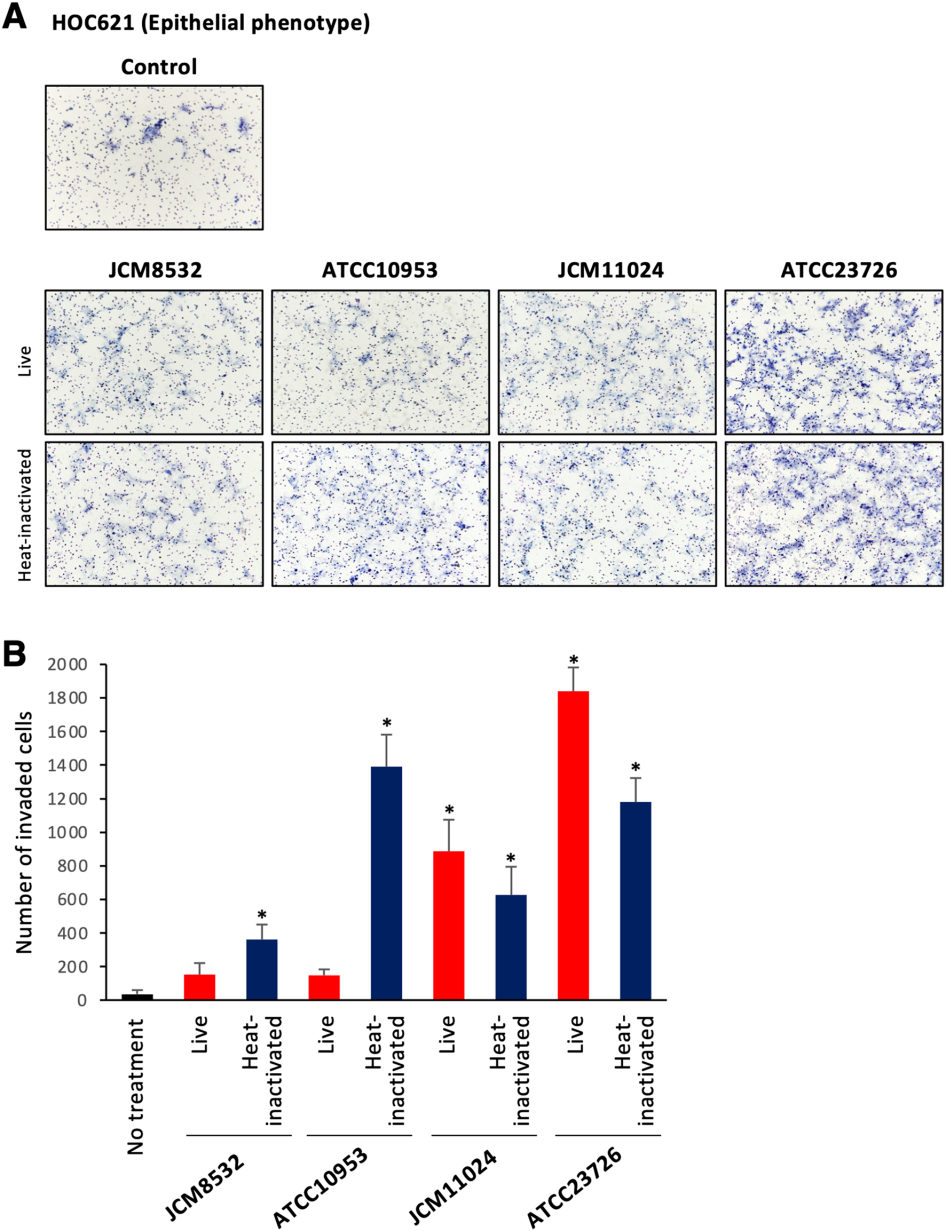
Invasive ability is promoted by co-culture with live or heat-inactivated *F. nucleatum* in epithelial phenotype, HOC621 cells. After co-culture with 4 subspecies of live or heat-inactivated *F. nucleatum* (MOI = 10), invasion ability was determined by in vitro invasion assay in epithelial phenotype, HOC621 cells. (**A**) Pictures showed the invaded OSCC cells after co-cultured with 4 subspecies of live or heat-inactivated *F. nucleatum.* (**B**) Graph shows the number of invaded OSCC cells after co-cultured with 4 subspecies of live or heat-inactivated *F. nucleatum*. Three independent experiments were used to calculate the mean ± S.D. (*) represents *P*-value of < 0.05 compared to control (no treatment).

It has been shown that p-EMT program in cancer cells enhances the invasive properties. Therefore, we examined the invasion ability of HOC621 cells after treatment with 4 subspecies of *F. nucleatum* (JCM8532, ATCC10953, JCM11024, and ATCC23726) by in vitro invasion assay. Most live or heat-inactivated *F. nucleatum* subspecies statistically promoted the invasion ability (Fig. 7A,B). Enhanced invasion ability by *F. nucleatum* was also observed in HSC2 cells (Supplementary Fig. S6). These findings indicate that *F. nucleatum* may enhance the invasion ability via upregulation of p-EMT-related genes in OSCC cells with epithelial phenotype.

**Figure 8:**
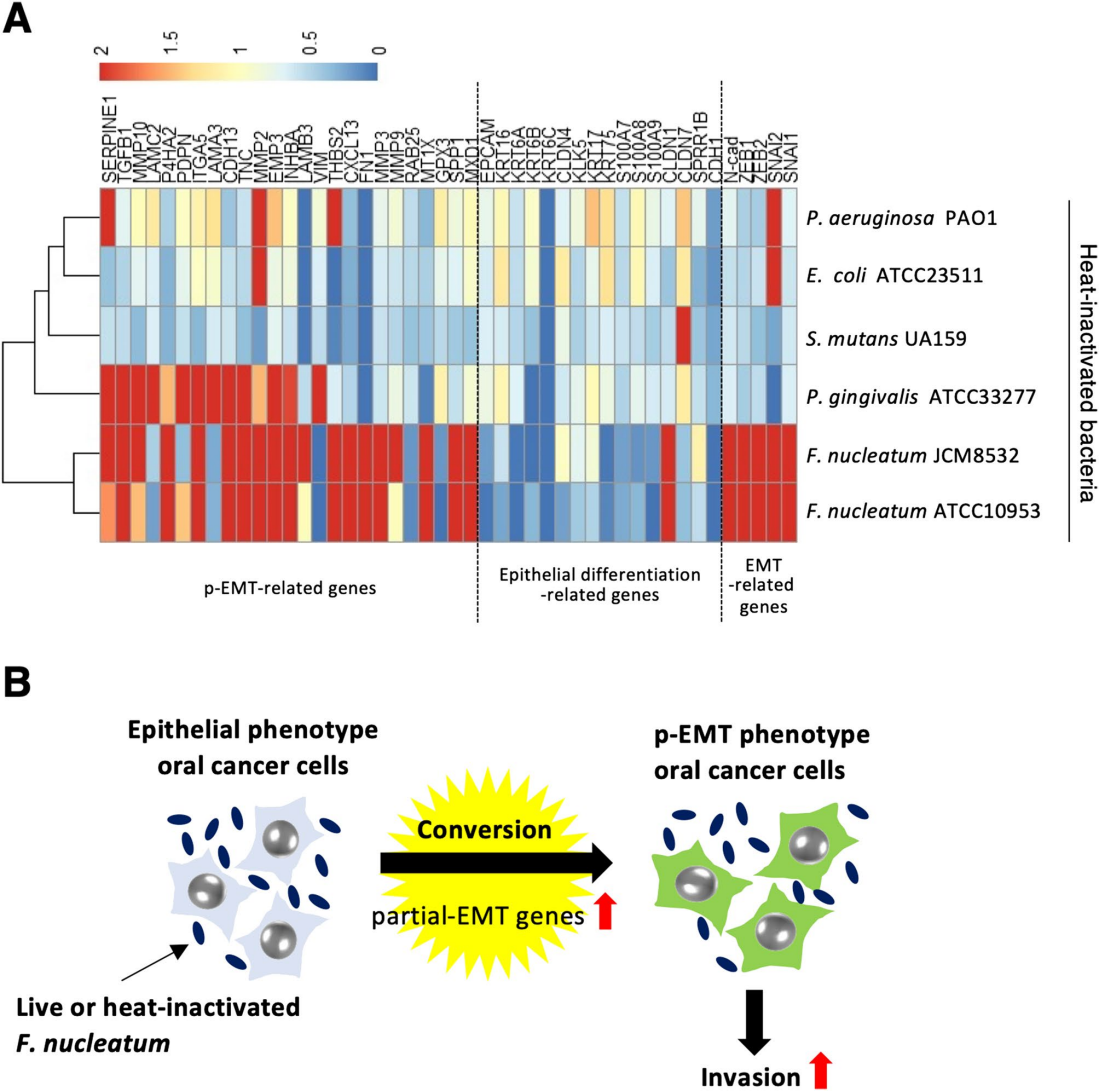
Remarkable upregulation of p-EMT-related genes by *F. nucleatum* in epithelial phenotype, HOC621 cells. (**A**) Heatmap shows the expression levels of epithelial differentiation-related, p-EMT-related, and EMT-related genes after co-culture with heat-inactivated *F. nucleatum*, *P. gingivalis*, *S. mutans*, *P. aeruginosa*, and *E. coli* in epithelial phenotype, HOC621 cells. Gene expression data is shown in Supplementary Fig. S8. (**B**) Schematic model showing that *F. nucleatum* promotes invasion of OSCC cells via conversion from epithelial to p-EMT phenotype.

To know whether upregulation of p-EMT-related genes in OSCC cells with epithelial phenotype is specific event by *F. nucleatum*, we examined the gene expression in HOC621 cells after treatment with other strains, such as *P. gingivalis* (ATCC33277), *S. mutans* (UA159), *P. aeruginosa* (PAO1), and *E. coli* (ATCC23511). These bacteria did not change the morphology of HOC621 cells (Supplementary Fig. S7A). *E. coli* and *S. mutans* did not affect cell proliferation, but *P. gingivalis* and *P. aeruginosa* suppressed cell proliferation (Supplementary Fig. S7B). Most bacterial cells, except *P. gingivalis*, did not affect the expression of p-EMT-related genes (Fig. 8A and Supplementary Fig. S8).

## Discussion

Bacteria, fungi, viruses, and archaea naturally colonize in the oral cavity. The oral microbiota refers to a highly varied and complicated ecosystem of these organisms. Over 700 bacterial species are endemic to the oral cavity, and oral commensal bacteria play a critical role in the maintenance of a normal oral physiological environment and the development of systemic diseases^23,24^. It recently has been shown that oral commensal bacteria may be involved in the pathogenesis of OSCC^25,26^. The proportion of anaerobes within the biofilms on the surfaces of OSCC tissues is higher than that in the healthy control, indicating that OSCC surfaces provide an important reservoir for anaerobic bacteria^27^. There are many reports showing that *Fusobacterium* were detected in OSCC tissues^28,29^. Moreover, *Fusobacterium* was enriched in OSCC biopsy compared to fibroepithelial polyp as a control^13^. Thus, distribution of bacteria including *Fusobacterium* in OSCC tissues may be distinct from that in healthy oral mucosal tissues. As shown in Fig. 2B, *Fusobacterium* can invade into OSCC cells. Previous report also shows that *F. nucleatum* adheres to and invades into human gingival epithelial cells (HGECs) and OSCC cells^30^. Interestingly, the spontaneous mutant of *F. nucleatum* isolated as defective in autoagglutination was unable to invade into HGECs and OSCC cells, suggesting the requirement for bacterial components to their invasion^30^. Moreover, glucose inhibition assay shows that lectin-like interactions are involved in the attachment of *F. nucleatum* to OSCC cells^30^. In the present study, both live and heat-inactivated *F. nucleatum* enhanced the invasion ability of OSCC cells via upregulation of p-EMT genes. Although some bacterial cells directly invaded into OSCC cells, most bacterial cells attached on the surface of the cells (Fig. 2B). Notably, heat-inactivated *F. nucleatum* as well as live *F. nucleatum* upregulated p-EMT-related genes and enhanced the invasive ability. These findings suggest that heat-resistant bacterial cell components may be involved in promoting invasion ability via upregulation of p-EMT genes. However, further experiments will be required for clarifying this mechanism.

In recent years, attention has been focused on the involvement of *F. nucleatum* in colorectal cancer^8,9,31^. In colon tissue of patients with colorectal cancer, increased amount of *F. nucleatum* was significantly detected, compared with healthy people^32,33^. Moreover, *F. nucleatum* colonies in primary colorectal cancer tissues were detected in metastatic liver tissues^34^. Thus, cumulative clinical evidence indicates that the enrichment of *F. nucleatum* may correlate with colorectal cancer metastasis^32,34–37^. Interestingly, the outer membrane proteins of *F. nucleatum*, *Fusobacterium* adhesion A (FadA) and Fibroblast activation protein 2 (Fap2) have been focused as a pathogen of colorectal cancer. FadA is required for bacterial attachment and invasion of gingival epithelial cells and endothelial cells^30^. FadA-mediated interaction with E-cadherin is involved in attachment and invasion into colorectal cancer cells^8^. Fap2 also plays a critical function in the development of colorectal cancer. Fap2 is involved in the inactivation of immune cells including T cells and NK cells via an immunoreceptor, TIGIT in colorectal cancer^31^. Moreover, *Fusobacteria* use a hematogenous route to reach colon adenocarcinomas via interaction between Fap2 and Gal-GalNAc^9^. We will examine the involvement of outer membrane proteins of *F. nucleatum*, FadA and Fap2 in the promotion of invasion via upregulation of p-EMT genes in OSCC cell with epithelial phenotype.

Recently, it has been shown that exposure of primary epithelial cell cultures to heat-inactivated *F. nucleatum* induces downregulation of E-cadherin and upregulation of N-cadherin, vimentin, Snail, matrix metalloproteinase-2 and toll-like receptor 4 (TLR-4) and promotes migration^38^. Another study shows that heat-inactivated *F. nucleatum* upregulates TLR-4 in oral keratinocyte^39^. TLR-4 is known to be responsible for recognition of LPS and activation of this pathway results in increased production of inflammatory cytokines^40^. Indeed, we showed that heat-inactivated *F. nucleatum* promoted invasion via upregulation of p-EMT genes in OSCC cells with epithelial phenotype. These findings suggest that *F. nucleatum* LPS may be involved in p-EMT and/or EMT induction via TLR signaling. Interestingly, previous studies show that LPS promotes EMT via activation of TLR4/JNK signaling^41,42^. Importantly, the effect of *F. nucleatum* on upregulation of the genes involved in invasion, survival, and EMT is comparable or greater than other periodontal bacteria, *P. gingivalis*, *Treponema denticola*, and *Tannerella forsythia*. In the present study, *S. mutans*, *P. aeruginosa*, and *E. coli* did not affect the expression of p-EMT-related genes. Interestingly, *P. gingivalis*, which is also known as periodontal pathogen, upregulated p-EMT genes in HOC621 cells (Fig. 8A). Cumulative evidences suggest that *F. nucleatum* and/or *P. gingivalis* among periodontal bacteria may have greater effects of promoting invasion, survival, and p-EMT/EMT during OSCC progression.

It is recognized that the ability of cancer cells to undergo p-EMT, rather than complete EMT, poses a higher metastatic risk^16^. Recent single cell transcriptome analysis in HNSCC identifies several genes involved in p-EMT program^43^. Among these p-EMT-related genes, we identified the prognosis-related genes, such as *SERPINE1*, *TGFBI*, *ITGA5*, *CDH13*, *P4HA2*, and *LAMC2*^18^. Thus, p-EMT program is correlated with poor prognosis of HNSCC. Importantly, the enrichment of *F. nucleatum* is frequently observed in OSCC tissues compared to healthy oral mucosal tissues. Conversion from epithelial to p-EMT phenotype via alteration of gene expression by *F. nucleatum* infection may lead to increase a metastatic ability of OSCC (Fig. 8B). As *F. nucleatum* is well known oral commensal bacterium involved in periodontal diseases, oral hygiene managements for reducing the amount of *F. nucleatum* may contribute to reduce the risk for an increase in metastatic ability of OSCC.

## Supplementary Information

Supplementary Information 1.

Supplementary Information 2.

Supplementary Information 3.

Supplementary Information 4.

Supplementary Information 5.

Supplementary Information 6.

Supplementary Information 7.

Supplementary Information 8.

Supplementary Information 9.
