# Supplementary figures and images for: Conversion from epithelial to partial-EMT phenotype by Fusobacterium nucleatum infection promotes invasion of oral cancer cells
Source: Sci Rep. 2021 Jul 22;11:14943. doi: 10.1038/s41598-021-94384-1 (PMC8298429; doi:10.1038/s41598-021-94384-1)

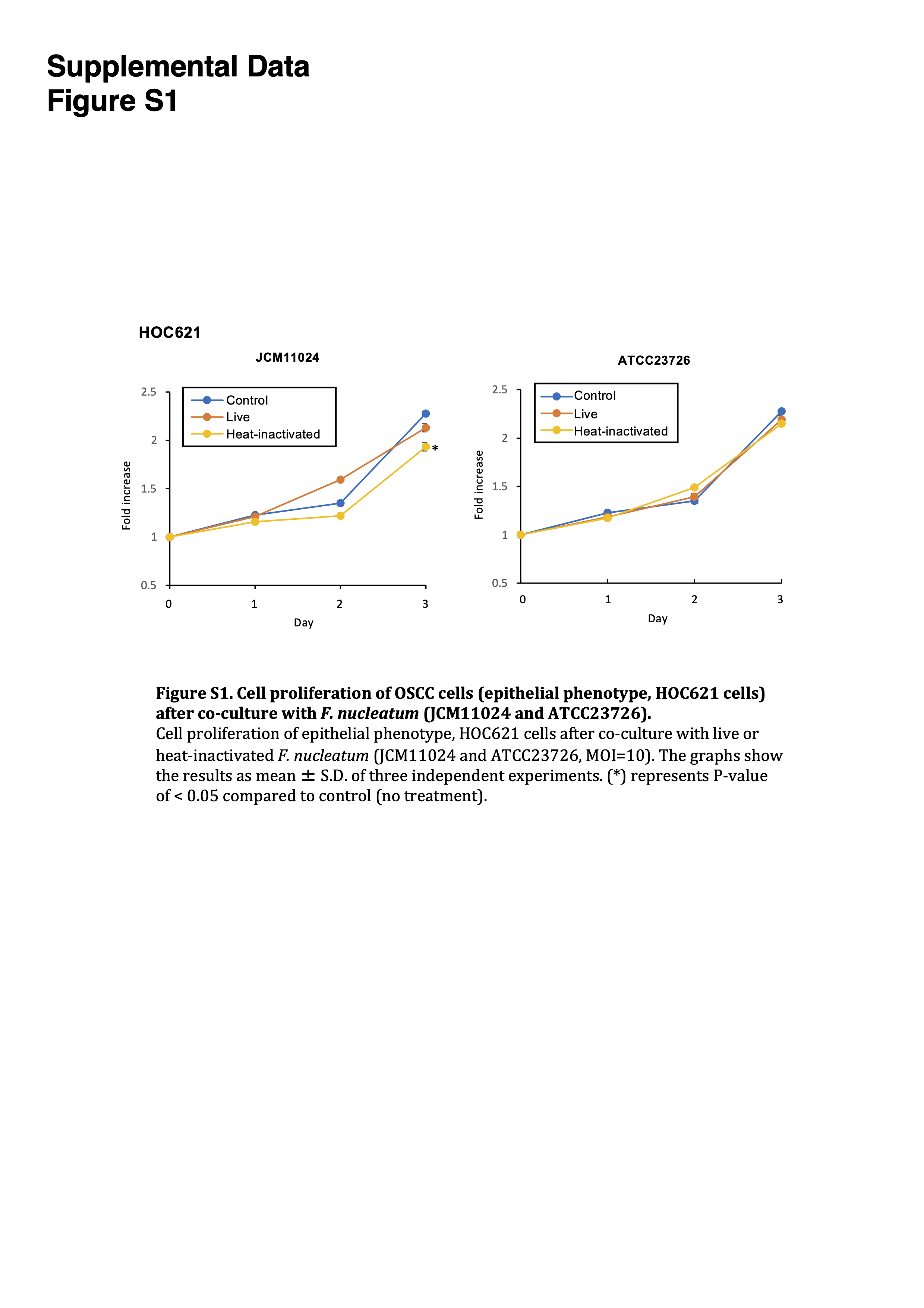

Supplement: Supplementary file 1 — Supplementary Information 1. [file 41598_2021_94384_MOESM1_ESM.tiff]

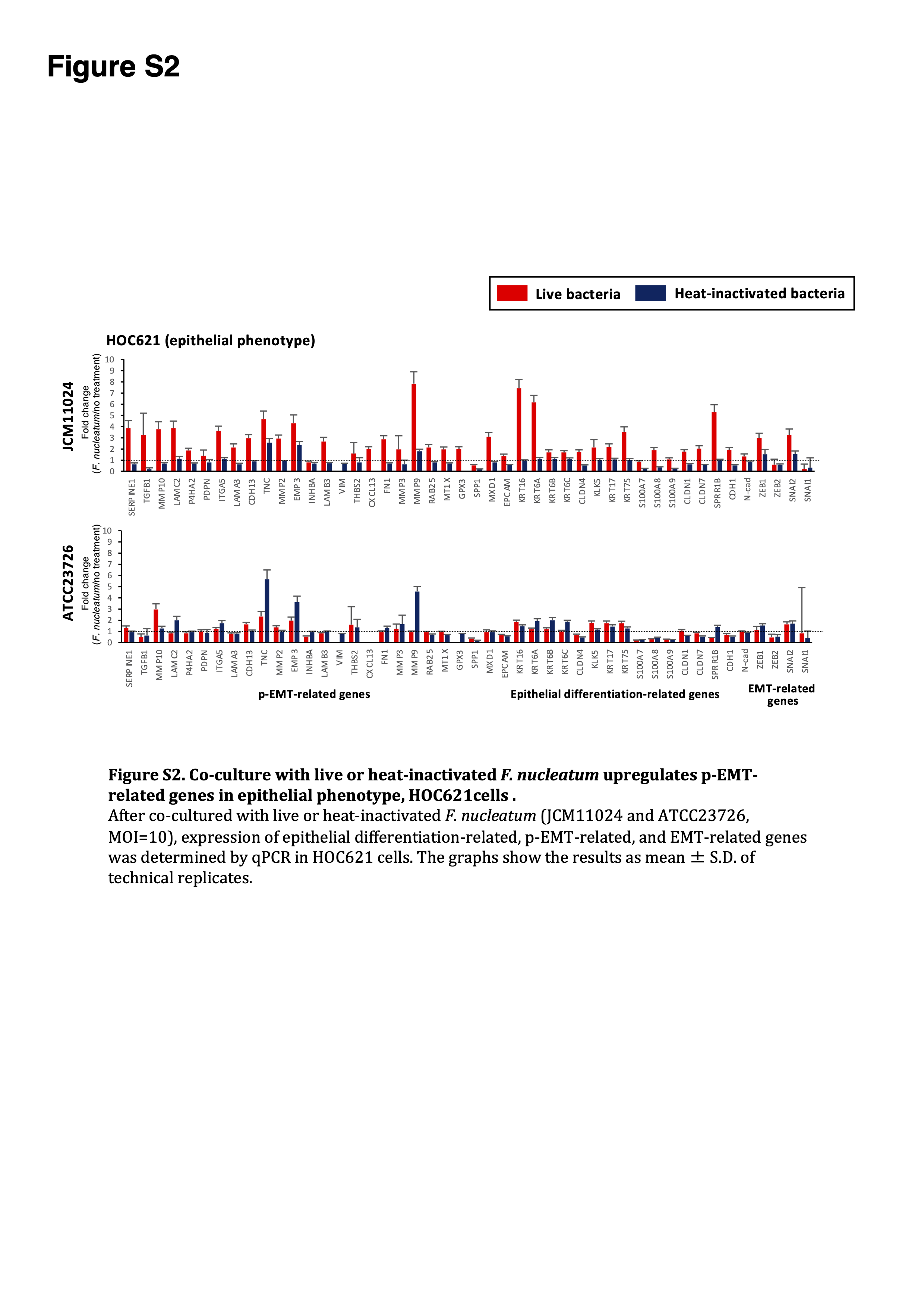

Supplement: Supplementary file 2 — Supplementary Information 2. [file 41598_2021_94384_MOESM2_ESM.tiff]

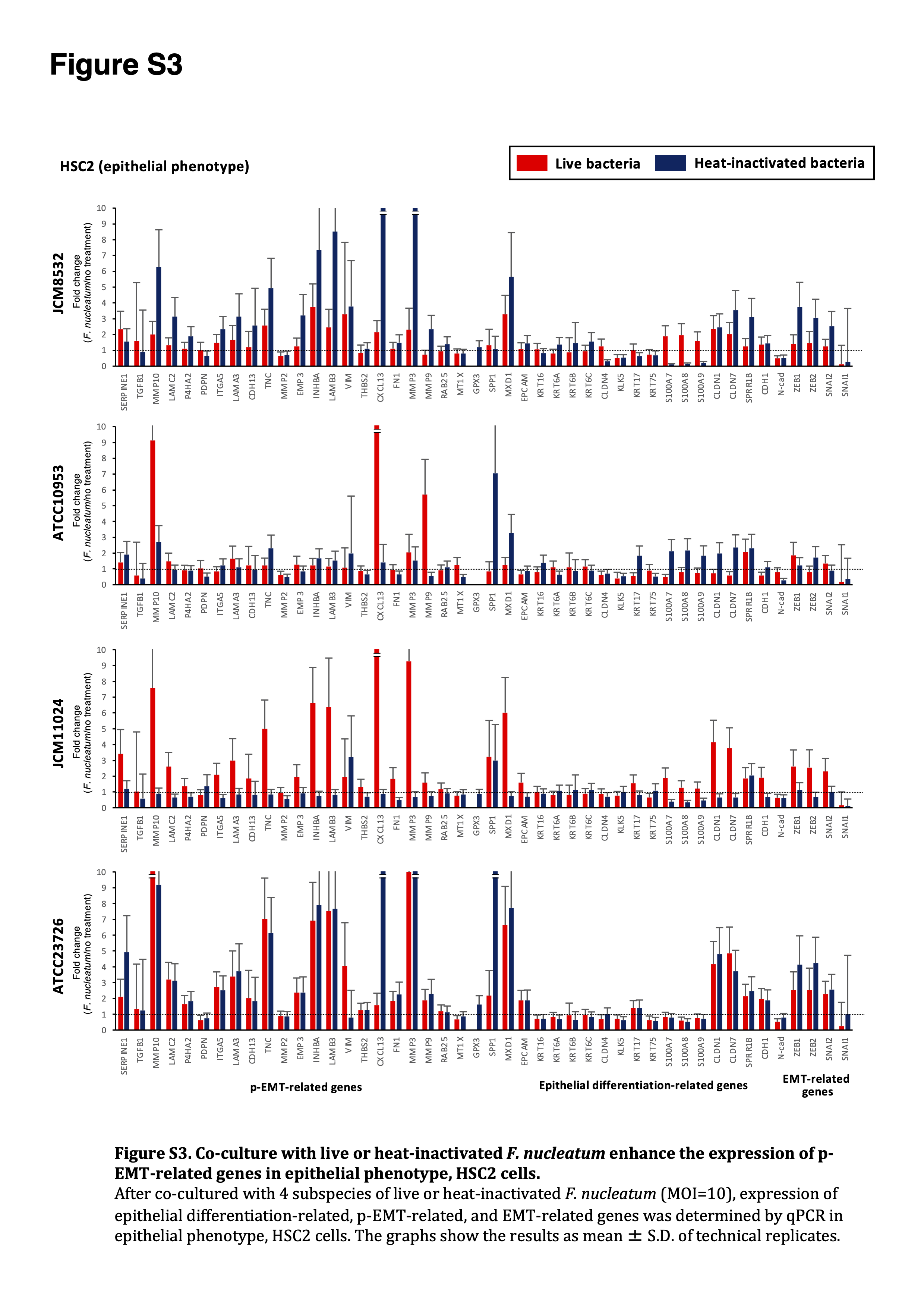

Supplement: Supplementary file 3 — Supplementary Information 3. [file 41598_2021_94384_MOESM3_ESM.tiff]

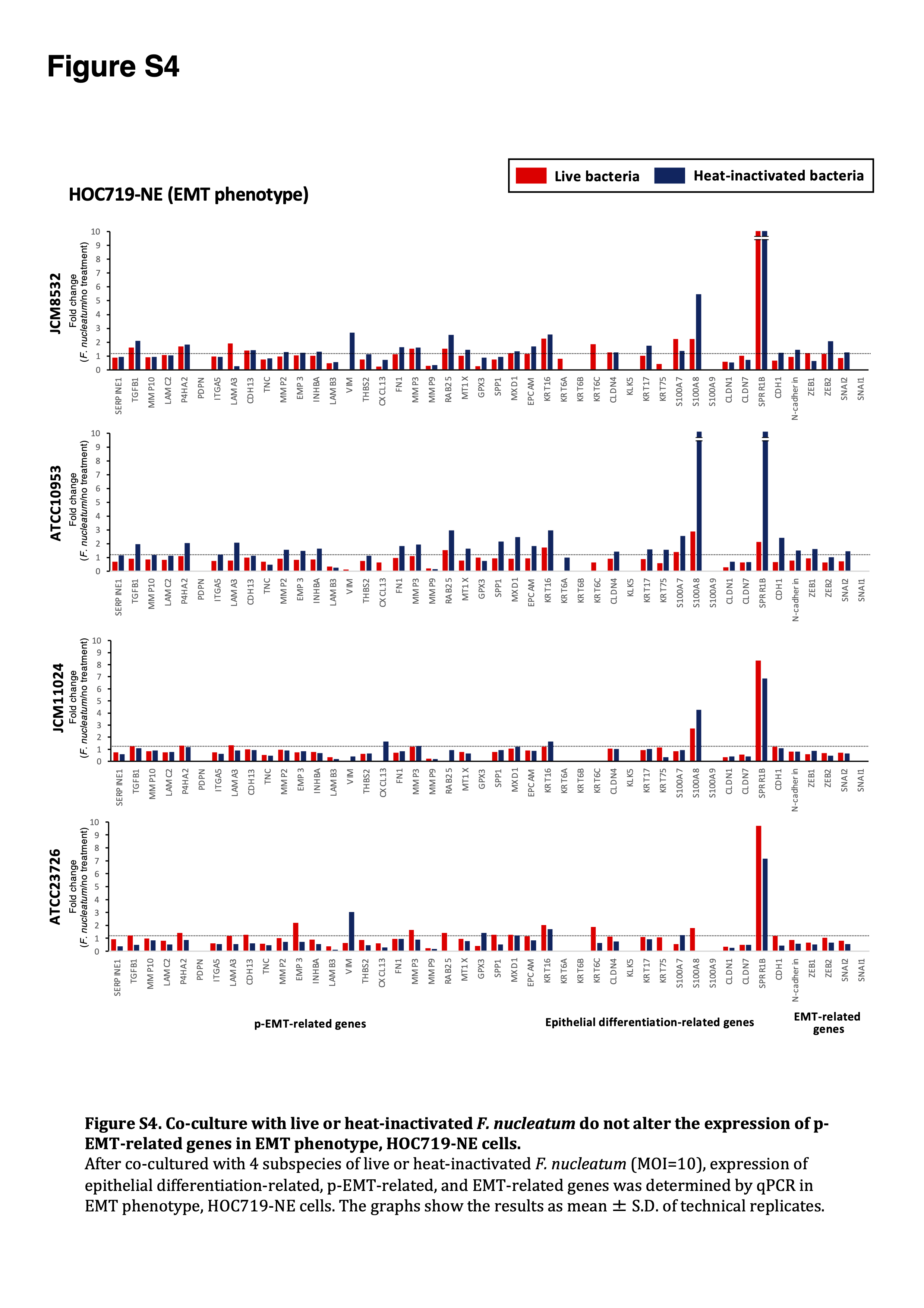

Supplement: Supplementary file 4 — Supplementary Information 4. [file 41598_2021_94384_MOESM4_ESM.tiff]

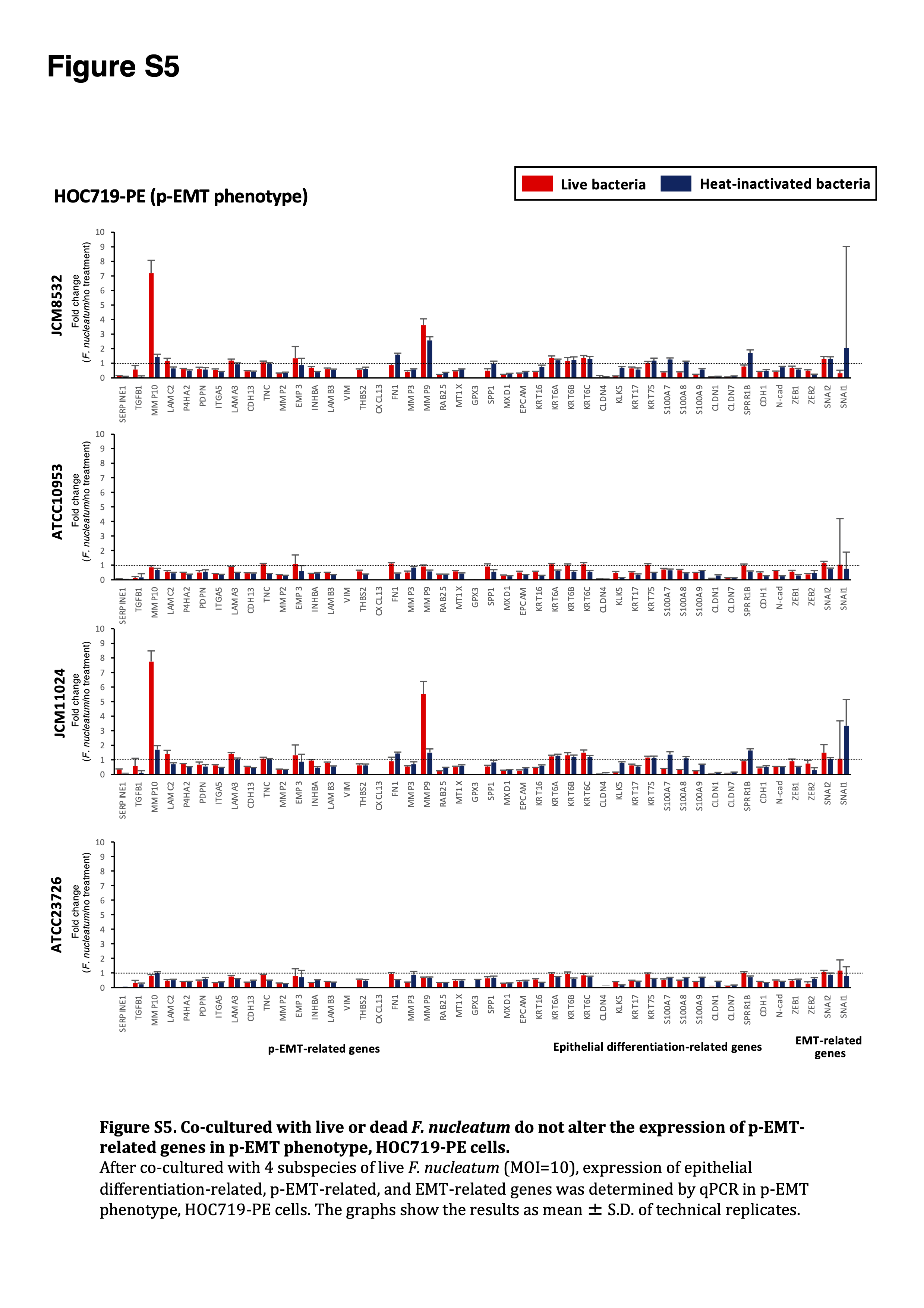

Supplement: Supplementary file 5 — Supplementary Information 5. [file 41598_2021_94384_MOESM5_ESM.tiff]

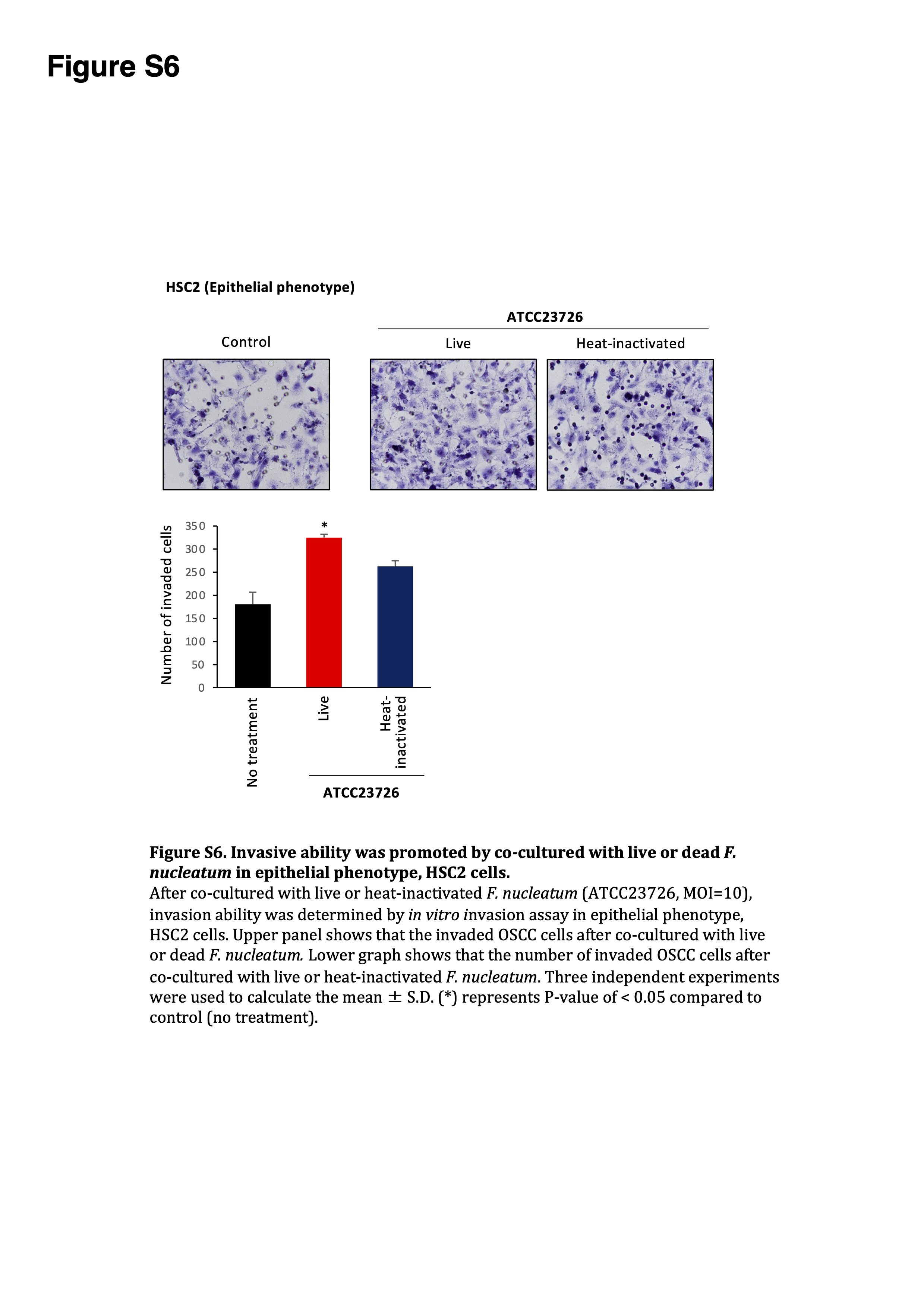

Supplement: Supplementary file 6 — Supplementary Information 6. [file 41598_2021_94384_MOESM6_ESM.tiff]

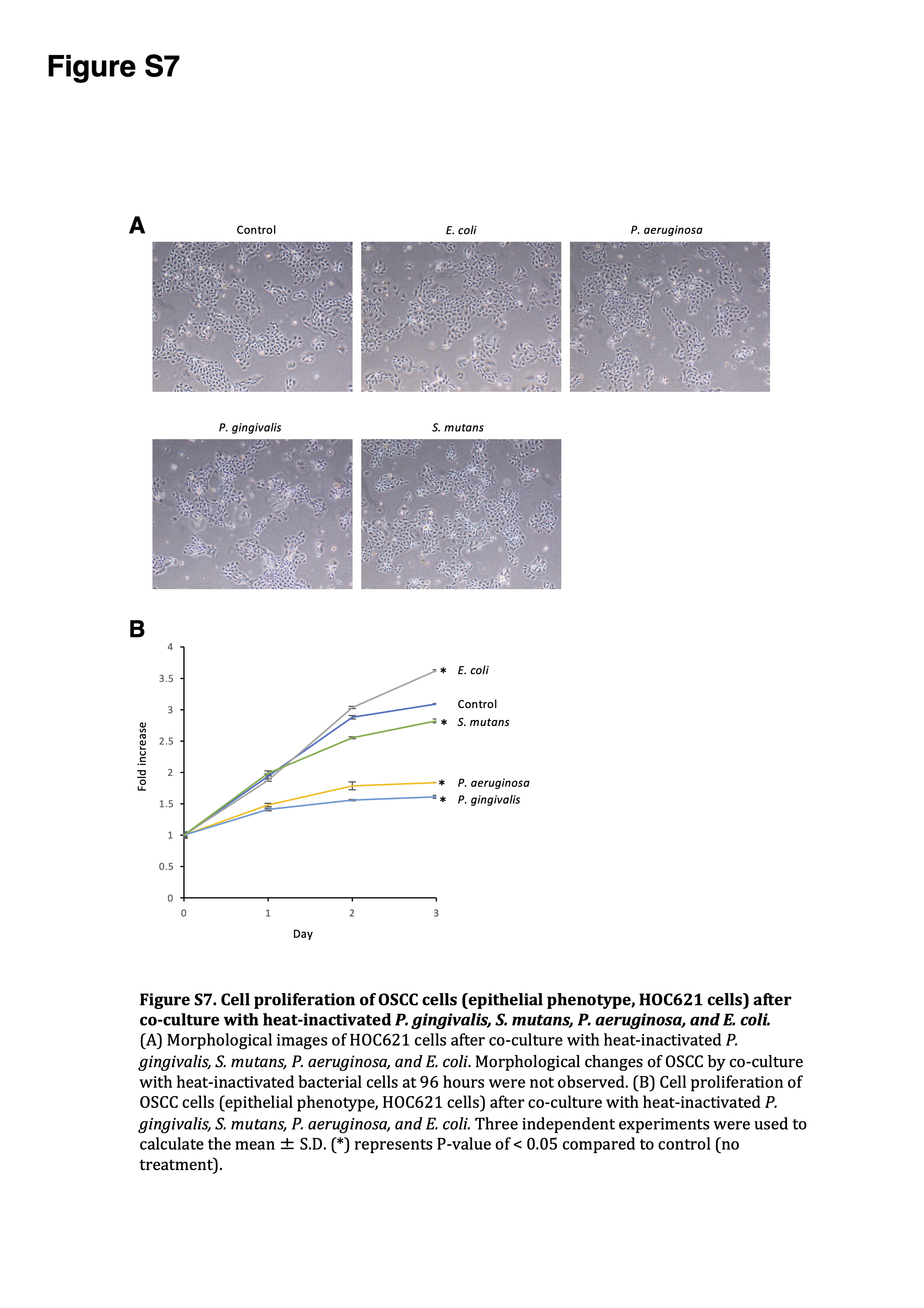

Supplement: Supplementary file 7 — Supplementary Information 7. [file 41598_2021_94384_MOESM7_ESM.tiff]

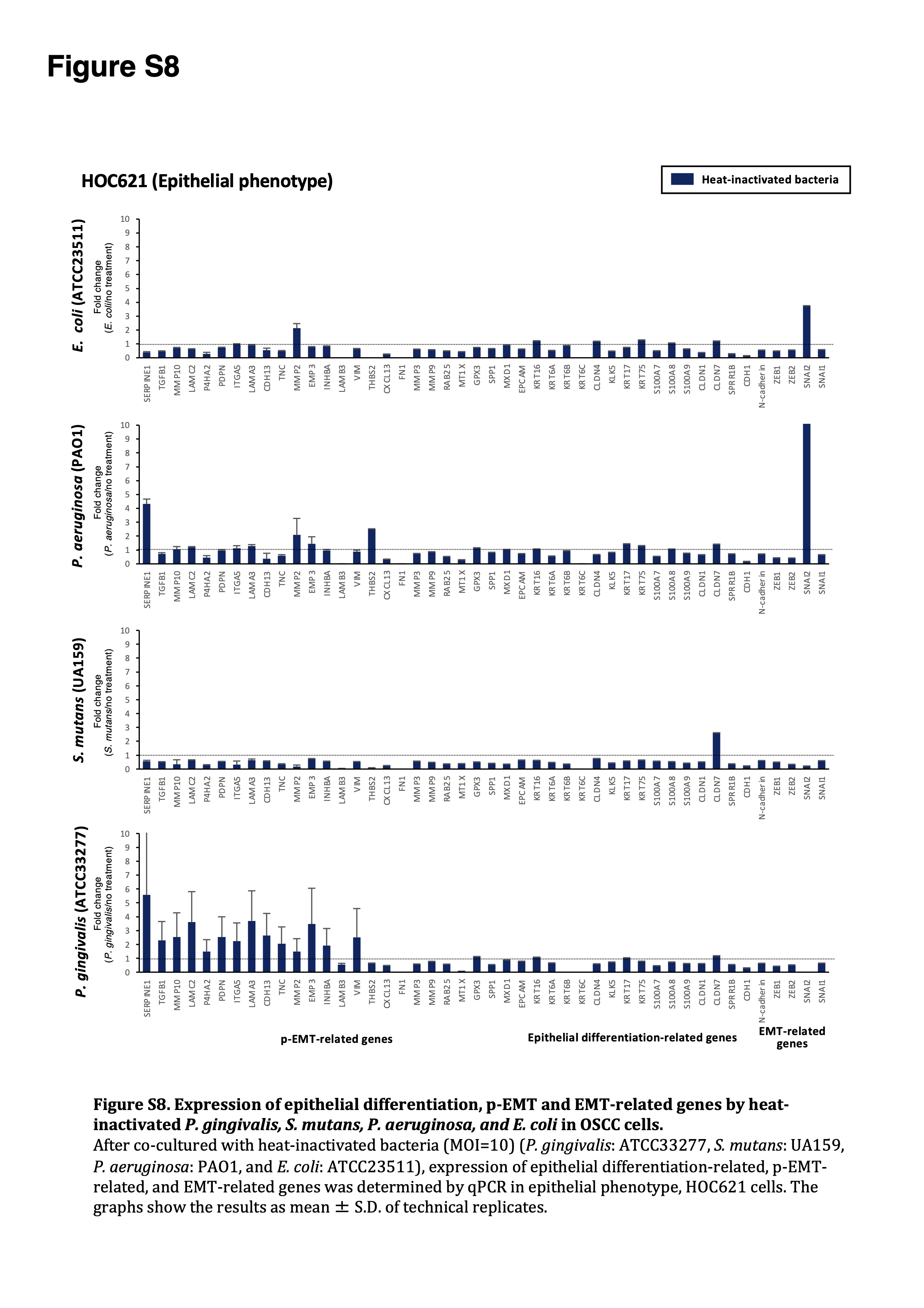

Supplement: Supplementary file 8 — Supplementary Information 8. [file 41598_2021_94384_MOESM8_ESM.tiff]

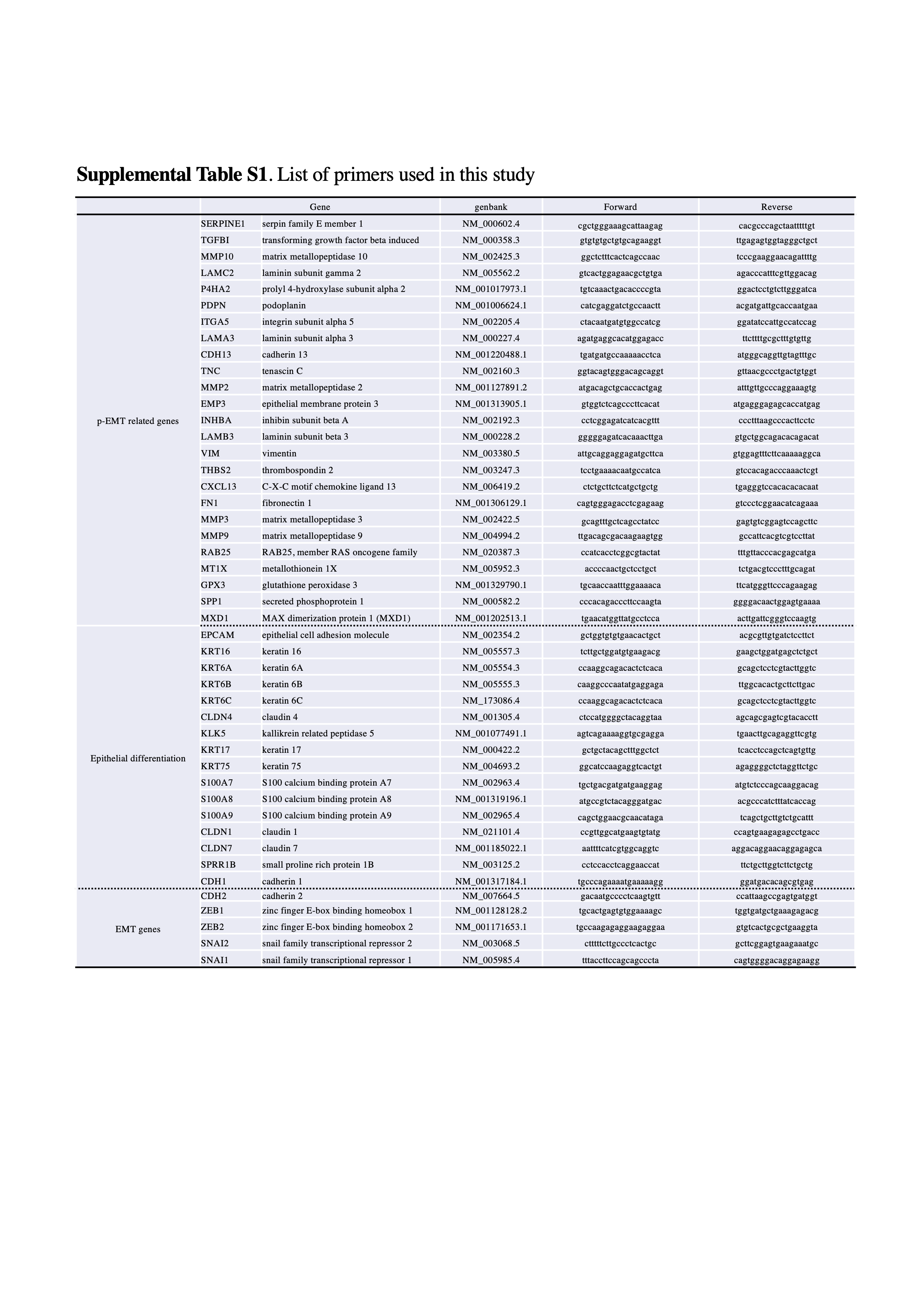

Supplement: Supplementary file 9 — Supplementary Information 9. [file 41598_2021_94384_MOESM9_ESM.tiff]
